# Empirical evidence for definitions of episode, remission, recovery, relapse and recurrence in depression: a systematic review

**DOI:** 10.1017/S2045796018000227

**Published:** 2018-05-17

**Authors:** P. L. de Zwart, B. F. Jeronimus, P. de Jonge

**Affiliations:** 1 University of Groningen, University Medical Center Groningen, Department of Psychiatry, Interdisciplinary Center Psychopathology and Emotion Regulation (ICPE), Groningen, The Netherlands; 2 University of Groningen, Faculty of Behavioural and Social Sciences, Department of Developmental Psychology, Groningen, The Netherlands

**Keywords:** Depression, evidence-based psychiatry, mood disorders unipolar, outcome studies, systematic reviews

## Abstract

**Aims.:**

For the past quarter of a century, Frank *et al.*’s (1991) consensus-based definitions of major depressive disorder (MDD) episode, remission, recovery, relapse and recurrence have been the paramount driving forces for consistency in MDD research as well as in clinical practice. This study aims to review the evidence for the empirical validation of Frank *et al.*’s proposed concept definitions and to discuss evidence-based modifications.

**Methods.:**

A literature search of Web of Science and PubMed from 1/1/1991 to 08/30/2017 identified all publications which referenced Frank *et al.*’s request for definition validation. Publications with data relevant for validation were included and checked for referencing other studies providing such data.

**Results.:**

A total of 56 studies involving 39 315 subjects were included, mainly presenting data to validate the severity and duration thresholds for defining remission and recovery. Most studies indicated that the severity threshold for defining remission should decrease. Additionally, specific duration thresholds to separate remission from recovery did not add any predictive value to the notion that increased remission duration alleviates the risk of reoccurrence of depressive symptoms. Only limited data were available to validate the severity and duration criteria for defining a depressive episode.

**Conclusions.:**

Remission can best be defined as a less symptomatic state than previously assumed (Hamilton Rating Scale for Depression, 17-item version (HAMD-17) ⩽4 instead of ⩽7), without applying a duration criterion. Duration thresholds to separate remission from recovery are not meaningful. The minimal duration of depressive symptoms to define a depressive episode should be longer than 2 weeks, although further studies are required to recommend an exact duration threshold. These results are relevant for researchers and clinicians aiming to use evidence-based depression outcomes.

## Introduction

Major depressive disorder (MDD) is a common, often chronic and recurrent condition, marked by persistent suffering and poor overall health and with deleterious effects on psychosocial, academic, vocational and family functioning. MDD is one of the most prevalent mental disorders and the leading cause of disability worldwide (World Health Organization, 2017), with lifetime prevalence estimates ranging from 7% to 21% (Kessler & Bromet, 2013).

**Fig. 1.**
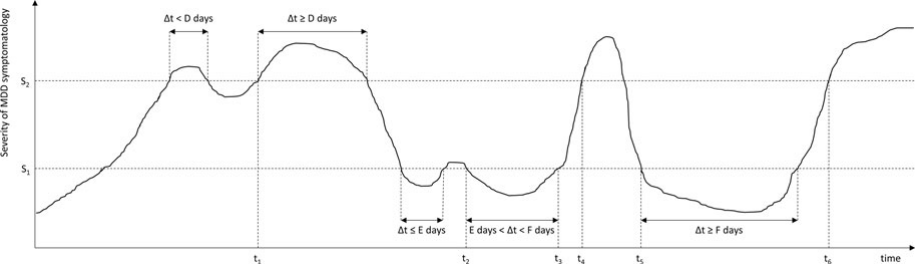
Time course of depressive symptomatology in a hypothetical patient, showing an MDD episode, remission, relapse, recovery and recurrence. These stages are operationalised using two severity criteria (*S*_1_, *S*_2_), and three duration criteria (*D*, *E*, *F*). *S*_1_: Severity threshold separating asymptomatic from partially symptomatic range; *S*_2_: Severity threshold separating partially symptomatic range from fully symptomatic range; *t*_1_, Start of MDD episode; *t*_2_, Start of episode remission; *t*_3_, End of episode remission; *t*_4_, Relapse of MDD episode; *t*_5_: Start of episode recovery and end of MDD episode; *t*_6_, Start of MDD recurrence.

In 1991, the MacArthur Foundation Network on the Psychobiology of Depression concluded that the randomness with which investigators referred to key changes in clinical status of individuals with depression led to considerable confusion in the literature (Prien *et al.* 1991). Subsequently, a task force was initiated to achieve consensus about the definition of key stages, change points and outcome definitions for MDD among clinical investigators and practicing clinicians. The resulting report by Frank *et al.* (1991) defined conceptualisations of an MDD episode, remission, recovery, relapse and recurrence (see Fig. 1 and supplementary Table) by a set of five parameters or thresholds: two severity scores (cut-offs for ‘asymptomatic’ and fully symptomatic ranges) and three durations (minimal consecutive time durations in the fully or a-symptomatic range before an episode, remission, or recovery can be declared).

Specific consensus-based recommendations for these thresholds were provided in Frank *et al.*’s (1991) report and revised in a follow-up report by Rush *et al.* (2006). Both reports explicitly requested empirical validations of these now widely used consensus-based definitions. Therefore, the present paper reviews the accumulated evidence over the past 27 years to validate the proposed conceptualisations and operationalisations and to provide suggestions for future avenues.

### Conceptual discussion

Here we focus on conceptualisations of MDD episode, remission, recovery, relapse and recurrence by Frank *et al.* (1991, see supplementary Table), which are based exclusively on severity (number/intensity) and duration of clinical symptoms, and each has its own rationale and clinical implications. An MDD episode means that illness is present and that treatment is indicated. When the state of remission (a relatively brief period without clinically relevant symptoms during or at the end of an episode) is reached, no intensified treatment regimen is required or justified. A recovery (a sustained period of absence of clinically relevant symptoms, i.e. a sustained remission) means that the episode has ended and treatment can be discontinued or aimed at preventing subsequent episodes. Relapse/recurrence imply a return of symptoms during remission/recovery, respectively, and indicate a need for treatment intensification. The implicit distinction between relapse and recurrence is that a relapse is thought to be a return of symptoms of an ongoing episode that was symptomatically suppressed, whereas a recurrence represents an entirely new episode.

Importantly, these concepts can only have the ascribed interpretations and treatment implications if they have substantial predictive value for a future course. For example, treatment is indicated for those experiencing an episode because they have a worse prognosis than those who are experiencing symptoms that do not meet episode criteria. Therefore, the operationalisations of these concepts (i.e. the choice of severity and duration thresholds) should be chosen in such a way that they have optimal prognostic significance.

In particular, it should be possible to distinguish remission from recovery (and therefore relapse from recurrence), which are different only in their duration, by a difference in prognosis. The hypothesis is that those in remission have not (yet) fully recovered from the latently present episode (i.e. they are still undergoing a healing process) and therefore have a relatively high relapse rate compared with those who recovered. Those who recovered have a low recurrence rate that is no longer dependent on the time since their last episode and equal to the incidence rate of a risk factor-comparable population who never experienced an episode. Similarly, in cancer research, ‘full remission’ is defined as the period during which any sign of the disease is lacking, but during which a patient is particularly vulnerable for a relapse of the tumour since latent disease might still be present. When the remission is of sufficiently long duration, the patient can be (retrospectively) considered to be recovered or ‘cured’ as the passing of even more time does not provide additional protection to disease recurrence, the risk of which is similar to the incidence risk of a comparable healthy population.

Some of the clinical status concepts that are the subject of this review are also defined in the Diagnostic and Statistical Manual of mental disorders (DSM-5; American Psychiatric Association, 2013) and the International Classification of Diseases (ICD-10; World Health Organization, 1993), as summarised in Table 1.

**Table 1.** Comparison DSM-5 and ICD-10 definitions for depression concepts

Minimal consecutive time duration:			DSM-5	ICD-10
Episode	D	Symptomatic range	2 weeks	2 weeks^a^
Remission	E	Asymptomatic range	2 months	Not explicitly defined^b^
Recovery	F	Asymptomatic range	Not explicitly defined^b^	Not explicitly defined^b^
Asymptomatic		Range cut-off	⩽2 symptoms to no more than a mild degree	‘Free from any significant mood symptoms’, not specified further
Symptomatic		Range cut-off	⩾1 out of 2 core symptoms and ⩾5 out of 9 total symptoms	⩾2 out of 3 core symptoms and ⩾4 out of 10 total symptoms

a If the symptoms are particularly severe and of very rapid onset, it may be justified to make the diagnosis after less than 2 weeks.

b Although the term ‘recovery’ is mentioned in the DSM-5 and ICD-10, it is not explicitly defined. ‘Relapse’ is not mentioned in DSM-5 and ICD-10, whereas a recurrent episode is defined in DSM-5 as a return of symptoms during a remission (i.e. equivalent to the concept of ‘relapse’ by Frank *et al.* (1991)) and in ICD-10 as a depressive episode separated from a previous episode by at least 2 months free from any significant mood symptoms.

## Methods/literature search

This systematic review largely adhered to PRISMA guidelines (Moher *et al.* 2009). To review empirical evidence regarding the definitions proposed by Frank *et al.* (1991), we searched both Web of Science and Pubmed for studies that referenced them without imposing language restrictions (see supplementary PRISMA flow diagram). Duplicates and non-obtainable studies were excluded. Based on title and abstract, studies were excluded that (i) did not focus on individuals with MDD, (ii) were non-empirical, (iii) were of study types not expected to be useful for the purpose of this review (see online supplement), or (iv) focused on the evaluation of some association or cause-effect relation between variables.

The remaining articles were scrutinised for data that could (in)validate at least one of Frank's definitions. Because severity related criteria were necessarily instrument-specific we focused on articles determining cut-offs on the HAMD-17 and the Montgomery-Åsberg Depression Rating Scale (MADRS), which are the most widely used instruments (Zimmerman *et al.* 2004*a*). Studies using different methodologies were included (see results section). Criteria to define state duration should be maximally predictive of remaining in that state (Frank *et al.* 1991). Therefore, we sought studies that show the remission/recovery and relapse/recurrence of depressive episodes over time (via survival curves or equivalent).

Two authors (PLdZ, BFJ) extracted data independently and resolved discrepancies through discussion and consensus. References of included articles were searched for additional relevant studies. The literature search was last updated on August 30, 2017.

## Results

The 1570 identified papers (supplementary eFigure) included 214 duplicates and 26 non-obtainable papers. The study selection criteria (as outlined above) reduced the number to 117 papers and yielded 49 additional records via reference checks. From these 166 papers, 110 were excluded based on the full-text assessment. Thus 56 studies covering 39 315 subjects were included, and summarised in Tables 2–5.

**Table 2.** Asymptomatic threshold (above) and fully symptomatic threshold (below): Comparisons with a gold standard

First author	Year	Sample, country^a^	Size (N)	♀ (%)	Age (range & M (s.d.) at *T*_1_)	Scale to determine cut-off	Gold standard for asymptomatic range/remission	Advised or implicated cut-off for asymptomatic range
Asymptomatic								
Hawley *et al*.	2002	Patients, GB	684	N/A	N/A	MADRS	CGI-S (using two different methods; CGI-S = 2 interpreted as ‘midpoint’ between remission and no remission)	MADRS <9 or <10
Zimmerman *et al*.	2004*d*	Patients, USA	303	62	M = 43 (13) *R* = 18–79	MADRS	Broad: SCOR-D ⩽2	MADRS ⩽9
							Narrow: SCOR-D = 1^b^	MADRS ⩽4^b^
Zimmerman *et al*.	2005	Patients, USA	303	62	M = 43 (13) *R* = 18–79	HAMD-17	Broad: SCOR-D ⩽2	HAM-D17⩽5
							Narrow: SCOR-D = 1^b^	HAM-D17⩽2^b^
Bandelow *et al*.	2006	Patients, DK/DE	1922	± 70	M = ± 40 (12)	MADRS	CGI-S ⩽2	MADRS ⩽11
							CGI-S = 1 (not at all ill)	MADRS ⩽5
Ballesteros *et al*.	2007	Patients, ES	113	81	M = 45 (13)	HAMD-17	CGI-S = 1	HAMD-17 ⩽7^c^
Riedel *et al*.	2010	Patients, DE	846	62	M = 46 (12)	HAMD-17, MADRS	CGI-S = 1 (=normal /min. S_*x*_)	HAMD-17 ⩽6 MADRS ⩽7
Romera *et al*.	2011	Patients, ES	292	77	M = 51 (15)	HAMD-17	SOFAS ⩾80	HAMD-17 ⩽5^d^
Leucht *et al*.	2013	Patients, GB	7131	62	M = 45 (15)	HAMD-17	CGI-S = 1	HAMD-17 ⩽5
							CGI-S ⩽2	HAMD-17 ⩽7
Sacchetti *et al*.	2015	Patients, IT	169	64	M = 46 (12)	HAMD-17	7i-SF-12 predicting better than poor functioning	HAMD-17 ⩽4
Fully symptomatic								
Leucht *et al*.	2013	Patients, GB	7131	62	M = 45 (15)	HAMD-17	CGI-S ⩾ 2	HAMD-17 ⩾ 7
							CGI-S⩾3	HAMD-17⩾13/14

CGI-S, Clinical Global Impression – Severity scale (1, very much improved; 2, much improved; 3, minimally improved; 4,  no change; 5, minimally worse; 6, much worse; 7=very much worse); HAMD, Hamilton Rating Scale for Depression (a.k.a. HRSD, HDRS); MADRS, Montgomery–Åsberg Depression Rating Scale; M, mean; min., minimal; N, number of participants; N/A, not available; R, range; s.d., standard deviation; SF-12 , 12-item short-form health survey; SOFAS, Social and Occupational Functioning Assessment Scale; *S*_*x*_, symptoms; *T*_1_, baseline.

a Country codes (ISO Alpha-2 and 3): DE, Germany; DK, Denmark; ES, Spain; IT, Italy; USA, United States of America.

b cut-off preferred by authors, typically because this subgroup scored better on psychosocial functioning.

c High value attributed to specificity.

d Equal value placed on sensitivity and specificity (AUC: 0.81).

**Table 3.** Asymptomatic threshold: Comparison with general population (above) or other comparison (below)

First author	Pub year	Sample, country	Size (N)	♀ (%)	Age (range & M (s.d.) at *T*_1_)	Scale to determine cut-off	Criteria for selecting optimal cutoff	Advised or implicated cutoff for asymptomatic range
Comparison with general population								
Zimmerman *et al.*	2004*a*		569	31	M = 34 (8)	MADRS^a^	Gen.pop. mean	⩽4
							Gen.pop. mean + 1 SD	⩽10, or upper limit of normal values
Zimmerman *et al.*	2004*b*		1014	51	M = 40 (12)	HAMD^a^	Gen.pop. mean	HAMD-17 ⩽4 (slightly higher than Gen.pop. mean)
							Gen.pop. mean + 1 SD	HAMD-17 ⩽7
							Gen.pop. mean + 2 SD	HAMD-17 ⩽10
Other comparisons								
Zimmerman *et al.*	2004*e*	Patients, USA	117	62	M = 43 (13), *R* = 18-79			
Zimmerman *et al.*	2007	Patients, USA	50	62	M = 43 (13), R = 18–73			
Zimmerman *et al*.	2012*a*	Patients, USA	140	68	M = 50 (13)			
Zimmerman *et al*.	2012*b*	Patients, USA	63	65	M = 48 (14)			
Zimmerman *et al*.	2012*c*	Patients, USA	140	68	M = 50 (13)			
Zimmerman *et al*.	2012*d*	Patients, USA	142	68	M = 49 (14)			

Gen.pop., general population; HAMD, Hamilton Rating Scale for Depression (a.k.a. HRSD, HDRS); MADRS,  Montgomery–Åsberg Depression Rating Scale; M, mean; min., minimal; N, number of participants; N/A, not available; *R*, range; s.d., standard deviation; *S*_*x*_, symptoms; *T*_1_, baseline. USA, United States of America.

a Based on a review of 10 studies for the MADRS and a review of 27 studies for the HAMD.

**Table 4.** Asymptomatic threshold: comparison of prognosis

First author	Pub year	Sample, country^a^	Size (N)	♀ (%)	Age (range & M (s.d.) at *T*_1_)	Definition for bad prognosis (e.g. relapse, recurrence, episode)	Cut-off that distinguishes between good and bad prognosis
Paykel *et al.*	1995	Patients, GB	57	61	*R* = 18–65	RDC MDD ⩾1 month	HAMD-17 ⩽7
Maier *et al*.	1997	Gen.pop. & patients, DE	400	N/A	N/A	DSM-3-R MDD	<3 DSM MDD *S*_*x*_
Riso *et al*.	1997	Patients, USA	90	56	M = 38 (10)	HAMD-17 ⩾14 for ⩾2 week	HAMD-17 ⩽6
Judd *et al.*	1998	Patients, USA	237	63	M = 40 (15)	MDD PSR 5 or 6 for ⩾ 2 week (claim to use RDC criteria)	MDD PSR = 1; authors argue recovery should be defined as PSR = 0^b^
Van Londen *et al.*	1998	Patients, NL	49	59	M = 45	DSM-3-R MDD ⩾ 1 month	MADRS <2 per *S*_*x*_
Fava *et al.*	1999	Patients, IT	40	N/A	N/A	N/A	N/A
Judd *et al*.	2000	Patients, USA	96	60	M = 40 (15)	Unclear	Unclear
Kanai *et al*.	2003	Patients, JP	82	59	M = 44 (15)	Subthreshold’ *E*_*x*_ (⩾3 DSM-4 *S*_*x*_ OR ⩾1 DSM-4 *S*_*x*_ that were graver than mild degree) for ⩾1 month	HAMD-17 ⩽1
Pintor *et al.*	2004	Patients, ES	138	68	M = 53 (16), *R*⩾18	HAMD-17 ⩾15 (Frank criteria)	HAMD-17 ⩽7
Taylor e*t al.*	2004	Patients, USA	153	65	M = ± 69	MADRS > 15	MADRS lower^c^
Nierenberg *et al.*	2010	Patients, USA	943	N/A	M = 40, *R* = 18–75	QIDS-SR16 ⩾11 (according to authors about HAMD-17 ⩾14)	No clear cut-off^d^
Romera *et al*.	2011	Patients, ES	292	77	M = 51 (15)	CGI-S increase ⩾2 points and DSM-4 MDD criteria	Future relapses were not significantly predicted by certain cut-offs, probably due to the small number of relapses.
Dunlop *et al*.	2012	Patients, USA	258	68	M = 42, R = 18–65	HAMD-17 >12 and a < 50% decrease from *E*_*x*_ *T*_1_ at 2 consecutive visits or at the last visit before discontinuation.	HAMD-17 ⩽3
Kiosses & Alexopoulos	2013	Patients, USA	152	60	M = 72 (7), *R* = 60–89	PSR score ⩾5^b^	LIFE-PSR ⩽2^b^
Peselow *et al*.	2015	Patients, USA	387	59	M= 32 (12), R = 16–77	Not clearly indicated; MADRS ⩾15 or meeting DSM-4 criteria for MDD.	MADRS ⩽8
Judd *et al.*	2016	Patients, USA	322	60	M = 40 (15), *R* = 17–76	PSR-MDD = 5/6 or PSR = 3^b^ for ⩾2 week	PSR = 1^b^

DSM, Diagnostic and Statistical Manual; E_x_., Episode; Gen.pop., General population; HAMD, Hamilton Rating Scale for Depression (a.k.a. HRSD, HDRS); LIFE-PSR, Longitudinal Follow-up Examination (LIFE) Psychiatric Status Rating Scale (PSR); MADRS, Montgomery–Åsberg Depression Rating Scale; M, mean; min., minimal; N, number of participants; N/A, not available; R, range; PSR, Psychiatric Status Ratings**; RDC, Research Diagnostic Criteria; s.d., standard deviation; *S*_*x*_, symptoms; *T*_1_, baseline; QIDS-SR16, Quick Inventory of Depressive Symptomatology 16-item self-rating scale.

a *Country codes* (ISO Alpha-2 and 3): DE, Germany; ES, Spain; GB, United Kingdom; IT, Italy; JP, Japan; USA, United States of America.

b *Psychiatric status ratings*: (1) asymptomatic (return to usual self); (2) residual/mild affective *S*_*x*_; (3) partial remission, moderate *S*_*x*_ or impairment; (4) marked/major *S*_*x*_ or impairment; (5) meets definite MDD criteria without prominent psychotic *S*_*x*_ or extreme impairment; (6) meets definite criteria with prominent psychotic *S*_*x*_ or extreme impairment.

c The authors state that relapse becomes less likely when the MADRS score is lower, but there is no single cut-off that has high sensitivity and specificity for predicting relapse: ‘This suggests that there is no particular cut-off that is sufficient to consider as ‘low enough’ to protect against future relapse, so the primary conclusion would be to strive for the lowest score possible’.

d No particular cut-off: those with a greater number of residual symptom domains (out of nine possible DSM-IV criterion symptom domains) had a greater probability of relapse.

**Table 5.** Definitions of duration thresholds for episode, remission and recovery of major depressive disorder

First author	Year	Sample, country code^a^	Size (N)	♀ (%)	Age (range & M (S.D.) at *T*_1_)	Estimated point of rarity	Episode	Remission	Recovery	Relapse	Recurrence
Duration thresholds for episode											
Eaton *et al*.	1997	Gen.pop., USA	71	75	*R* = 18–70		Sadness/anhedonia & 2 other *S*_*x*_	N/A	≥1 year without depressive *E*_*x*_	N/A	First *E*_*x*_ after recovery
Spijker *et al.*	2002	Gen. pop., NL	250	67	*R* = 18–64		DSM-3-R def. via CIDI	No/min. depressive *S*_*x*_ on LCI for 3 month (US NIMH def. +1 month)	No distinction provided between remission and recovery	N/A	N/A
Wakefield & Schmitz	2013	Patients, USA	88	73	*R* = 18–98, M = 37		≥2 week sadness & ≥4 *S*_*x*_ of adequate severity	*E*_*x*_ before *T*_1_, but not at *T*_1_ interview	N/A	N/A	Remitted at *T*_1_, but *E*_*x*_ *T*_1_–*T*_2_.
Whiteford *et al*.	2013	Patients, USA	749	73	M = 34		Differs per study	Differs per study	N/A	N/A	N/A
Duration thresholds for remission and recovery											
Maj *et al.*	1992	Patients, IT	72	58	M = 42 (7), *R* = 27–55		RDC after interview with SADS-L	N/A	≥8 week absence of prominent dysphonic mood (RDC MDD crit. A) and presence ≤2 *S*_*x*_ MDD crit. B (each HAMD ≤1)	N/A	MDD *E*_*x*_ after recovery
Shea *et al*.	1992	Patients, USA	78	N/A	*R* = 21–60	N/A	RDC for MDD *E*_*x*_	N/A	Stable MDD *S*_*x*_ remission, requiring LIFE-II-PSRs ≤2^b^ (min./no *S*_*x*_) ≥8 week following treatment	2 week of meeting RDC for MDD (PSR ≥5^b^) after recovery	No distinction provided between relapse and recurrence.
Paykel *et al.*	1995	Patients, USA	57	61	N/A	±10 month	RDC MDD *D*_*x*_	2 month *S*_*x*_ below MDD criteria (retrosp. ass.)	N/A	Return to RDC MDD ≥ 1 month (retrosp. ass.)	N/A
Eaton *et al*.	1997	Gen.pop., USA	80	62	*R* > 18		LCI MDD *E*_*x*_ [1], i.e., sadness/anhedonia & ≥2 other *S*_*x*_	N/A	1 yearr in which there was no MDD *E*_*x*_	N/A	First *E*_*x*_ after recovery
Emslie *et al*.	1997	Patients, USA	59	46	M = 13 (3), *R* = 7–17		≥14 days MDD K-LIFE rating ≥5	≥14 days MDD K-LIFE rating ≥1	MDD K-LIFE rating ≥1 ≥60 days	*E*_*x*_ MDD after remission	*E*_*x*_ MDD after recovery
Flint & Rifat	1997	Patients, CA	84	64	M = 74, *R* = 60–80		DSM-3-R criteria for non-bipolar, non-psychotic MDD & HAMD ≥16	Point of response (HAMD ≤10) followed by ≥2 week of HAMD ≤10	Not explicitly def., but deducible.	DSM-3-R MDD criteria ≥1 week & HAMD ≥16 *within* 16 week after MDD remission	Same as relapse but ≥16 week of remission without relapse
Riso *et al*.	1997	Patients, USA	90	56	M = 38 (9.7)	±3 month	Not def., but inclusion RDC & HAMD ≥14	3 week HAMD ≤6	≥6 month HAMD ≤6	≥2 week after a response HAMD≥ 14 [2]	2 week HAMD ≥14 after 6 month of recovery
Judd *et al*	1998	Patients, USA	237	63	M = 40 (15)	±29–37 month	RDC: >2 week PSR-MDD 5 or 6^b^	No def. Period PSR-MDD 1–2^b^ before recovery	RDC >8 week MDD PSR = 1^b^ (asymptomatic recovery) or 2^b^ (residual recovery)	>2 week MDD PSR 5 or 6^b^ (RDC def.)	>2 week MDD PSR 5 or 6^b^ (RDC def.)
Kessing *et al*.	1998	Patients, DK	17447	66	56		Period of hospitalisation, ending when not readmitted 8 week after discharge	When discharged from hospital. Remission ends ≥8 week, when recovery starts.	>8 week after being discharged from the hospital	Readmission to hospital ≤8 week after discharge (within remission period)	Readmission to hospital >8 week after discharge (within recovery period)
Van Londen *et al*.	1998	Patients, NL	49	59	45	±4 month	DSM-3-R criteria for MDD	2 month S_*x*_ below DSM-3-R MDD threshold (MADRS<2). Partial remission MADRS <10 (1 MADRS *S*_*x*_ = 3 allowed if rest <3)	Full remission ≥ 6 month	≥1 month return to MDD DSM-3-R, before recovery	Recurrence defined as relapse, but during recovery.
Van Weel-Baumgarten *et al.*	1998	Patients, NL	222	61	*R* = 0–80		Day first MDD *D*_*x*_	No def., *E*_*x*_ ends as end MDD in case record, or 3 month without *S*_*x*_	N/A	N/A	New code or *S*_*x*_ description ≥3 month without such *S*_*x*_
Mueller *et al.*	1999	Patients, USA	380	61	M = 38		No explicit def.	N/A	The first of 8 wk of no/min. *S*_*x*_ (defined as PSR= 1 or 2). Before recovery PSR = 1–6	N/A	No explicit def. String of MDD PSR ratings used for course estimate
O'Leary *et al*.	2000	Patients, IE	85	57	M = 41		ICD-10 def. for *E*_*x*_ or recurrent depression & HAM-D ≥17. Recurrent *E*_*x*_ only HAM-D ≥17	≥2 week HAM-D <8	No focus on recovery	2 week re-appearance of HAM-D scale ≥17 *within* 6 month of remission onset	N/A
Solomon *et al*.	2000	Ex-patients, USA	318	59	M = 39		*D*_*x*_ made according to RDC (not further specified)	N/A	≥8 week no MDD *S*_*x*_ or ≤2 *S*_*x*_ at mild level (PSR)^b^	N/A	Reappearance of RDC MDD criteria ≥2 week after being recovered from preceding *E*_*x*_
Heinze *et al*.	2002	Patients, MX	228	85	N/A	±12 month	DSM (not further specified)	Unclear	Unclear	Unclear	Unclear
Kanai *et al.*	2003	Patients, JP	82	59	M = 44 (15)	±12 month	DSM-4 MDD	No focus on remission	NIMH CDS def.; 2 month with ≤2 mild MDD *S*_*x*_	No focus on relapse. [3]	DSM-4 MDD
Kennedy *et al*.	2003	Patients, GB	65	61	M = 41, *R* = 20–65	±5 month	RDC (LIFE and PSR at *T*_2_)	N/A	≥8 week asymptomatic (≤2 *S*_*x*_ RDC)	N/A	New *E*_*x*_ RDC MDD after recovery
Birmaher *et al*.	2004	Patients, USA	68	43	M = 11, *R* = 8–16		Inclusion when MDD according to DSM-3-R	N/A	No MDD ≥2 month, based on K-SADS-E or SADS-L (depending on age). No cut-off def.	N/A	Emergence of MDD *S*_*x*_ during recovery period (K-SADS-E or SADS-L). No cut-off def.
Pintor *et al.*	2004	Patients, ES	138	68	M = 53 (16), *R*≥18		Frank's criteria applied using the HDRS	Frank's criteria applied using the HDRS	N/A	Frank's criteria applied using the HDRS	N/A
Mattisson *et al.*	2007	Patients, SE	344	68	*R* = 20–83		Retrospective report on *E*_*x*_, with medium degree of impairment required.	No explicit def.	No explicit def.	No explicit def.	No explicit def. *E*_*x*_ after an earlier *E*_*x*_ with a ‘well period’ in-between. [4]
Naz *et al*.	2007	Patients, USA	87	59	M = 31	±14 month	DSM-4 D_*x*_ by a team of psychiatrists, using all available information (e.g., SCID).	≥8 week asymptomatic (only min. *S*_*x*_). Partial remission: some persistent *S*_*x*_ but not meeting MDD criteria. Cut-offs unclear.	N/A	DSM-4 *E*_*x*_ after achieving remission. Partial relapse >min. *S*_*x*_ but not fulfilling criteria *E*_*x*_.	N/A
Holma *et al.*	2008	Patients, FI	163	78	M = 42	±18 month	DSM-4 criteria for MDD based on interviews using graphic life charts.	≥2 month without fulfilling DSM-4 MDD criteria. Full remission when none of the 9 core *S*_*x*_ was rated. Partial remission ≤4 *S*_*x*_.	N/A	2 week return to DSM-4 MDD *within* 2 month after being below threshold	Return to *E*_*x*_ MDD after ≥2 month of partial/full remission
Yiend *et al.*	2009	Patients, GB	37	81	M = 35 (12)		DSM and RDC criteria, but test remains unclear.	DSM and RDC criteria, but test remains unclear.	≥3 month with a PSR of 1 or 2^b^	No distinction between relapse and recurrence	No distinction between relapse and recurrence
De Jonge *et al.*	2010	Patients, NL	267	64	M = 43 (11)	±9 month	DSM-4 criteria using CIDI	No distinction between remission and recovery	Defined as any period between MDD *E*_*x*_	No distinction between relapse and recurrence	*E*_*x*_ during recovery
O'Leary *et al*.	2010	Patients, IE	86	52	M = 38	±2 month	DSM-4 MDD (single or recurrent) & HAM-D17 ≥17	2 week HAM-D <8	N/A	Reappearance of *S*_*x*_ *within* 6 month of remission onset & 2 week HAM-D17 ≥17 and meeting DSM-4 MDD criteria	N/A
Dunlop *et al*.	2012	Patients, USA	258	68	M = 42, *R* = 18–65		DSM-4-TR by SCID	N/A	4 def. were tested based on 2 different severity criteria (HAMD-17≤7 or HAMD-17≤3) and 2 different duration criteria (≥8 week i.e. 56 days or ≥4 month i.e. 120 days)	N/A	HAM-D17>12 and <50% decrease from acute phase *T*_1_ at 2 consecutive visits or last visit before discontinuation. This definition does not correspond to *E*_x_ definition.
Martínez-Amorós *et al*.	2012	Patients, ES	127	66	65		DSM-4-TR	HAMD-21 ≤6; no duration criterion def.	No def., but can be deduced from other def.	Reappearance of MDD within 6 month after remission	Emergence of a new MDD *E*_*x*_ ≥6 month (presumably after remission)
Kiosses & Alexopoulos	2013	Patients, USA	152	60	M = 72 (7), *R* = 60–89	±15 month	Not clearly def. but can be understood to be PSR ≥5	PSR≤2 for 3 week, without depressed mood/anhedonia, after MDD *Ex*. [5]	Not specified, but can be deduced from other def.	PSR ≥5 during the first 6 month after remission	PSR ≥5 between 6 month and 2.5 yrs. after remission (last observation since *T*_1_)
Seemüller *et al*.	2014	Patients, DE	458	66	*R* = 25–65		Not def.	HAMD-17 ≤7	N/A	Rehospitalisation, suicide or suicide attempt with the explicit suicidal intention.	N/A
Peselow *et al*.	2015	Patients, USA	387	32	M = 32 (12), *R* = 16–77		DSM-4 MDD as administered by psychiatrist via interview.	MADRS ≤8		Relapse and recurrence not distinguished	Any return of *S*_*x*_ i.e., MADRS ≥15 or DSM-4 criteria for MDD. No distinction provided between relapse and recurrence
Judd *et al*.	2016	Patients, USA	322	60	M = 40 (15), *R* = 17–76		RDC criteria ≥2 week with ≥5 *S*_*x*_ including intense sadness or dysphoria		Various def. of recovery were compared, differing on severity (PSR = 1 vs. 2)^b^ and duration (4 or 8 week) [6]	Both relapse and recurrence def. as first of 2 week with syndromal MDD *S*_*x*_ (PSR = 5 or 6)^b^ or minor depression (PSR = 3)^b^.	Both relapse and recurrence def. as first of 2 week with syndromal MDD *S*_*x*_ (PSR = 5 or 6) or minor depression (PSR = 3)^b^.

ass., assessment; DSM, Diagnostic and Statistical Manual; CIDI, Composite International Diagnostic Interview; D_*x*_, diagnosis; def., definition; E_*x*_., Episode; Gen.pop., General population; HAMD, Hamilton Rating Scale for Depression; LCI, Life chart interview; M, mean; MDD, Major Depressive Disorder or unipolar depression; min., minimal; N, number of participants; N/A, not available; NIMH, National Institute of Mental Health; PSR, Psychiatric Status Ratings^b^; *R*, range; RDC, Research Diagnosis Criteria; retr., retrospectively; SADS-L, Schedule for Affective Disorders and Schizophrenia-Lifetime interview; s.d., standard deviation; S_*x*_, symptoms; *T*_1_, baseline wave; *T*_2_, follow-up wave.

a *Country codes* (ISO Alpha-2 and 3): CA, Canada; DE, Germany; DK, Denmark; ES, Spain; FI, Finland; GB, United Kingdom; IE, Ireland; IT, Italy; JP, Japan; MX, Mexico; NL, Netherlands; SE, Sweden; USA, United States of America.

b *Psychiatric status ratings:* (1) asymptomatic (return to usual self); (2) residual/mild affective *S*_*x*_; (3) partial remission, moderate *S*_*x*_ or impairment; (4) marked/major *S*_*x*_ or impairment; (5) meets definite MDD criteria without prominent psychotic *S*_*x*_ or extreme impairment; (6) meets definite criteria with prominent psychotic *S*_*x*_ or extreme impairment.

(1) Respondents rated whether they experienced ‘a time when you felt sad or blue and had some of these other problems (e.g., weight loss or sleeplessness)’.

(2) Response was defined in various ways, and each definition was tested for validity.

(3) Authors appear to mix up recurrence and relapse, but we denote time after patient recovered as recurrence.

(4) Medication use was seen as indication for not being healthy, thus these people were not at risk for recurrence.

(5) PSR ≥3 during some of these weeks count as residual *S*_*x*_ after remission, i.e., the patient is not yet considered to be relapsed or recurred before PSR ≥5.

(6) The authors suggest that 8 week duration was the standard before their paper was published, mistakenly, see Rush *et al*. (2006).

### Severity thresholds

Frank *et al.* (1991) categorised the level of MDD symptomatology in three clinical ranges: a *fully symptomatic range* that can indicate the start of an episode, an *asymptomatic range* that can indicate the start of a full remission, and a *partially symptomatic range* in between. The ‘asymptomatic range’ is supposed to represent the normal range consistent with the absence of disorder. The term is a bit of a misnomer as this range includes the presence of a minor level of symptomatology associated with the ‘healthy’ (non-depressed) population, in which the average HAMD-17 score is about 3.2 (Zimmerman *et al.* 2004*b*); however, for consistency, the term asymptomatic will be used throughout this review.

Two instrument-specific ‘thresholds’ need to be defined on the HAMD-17 and MADRS (most widely used as endpoints in clinical trials; Zimmerman *et al.* 2004*a*) to operationalise these three different levels of symptomatology (see Fig. 1). Frank *et al.* (1991) defined HAMD-17 scores ⩾15 to correspond to the fully symptomatic range while HAMD-17 ⩽7 would indicate the asymptomatic range, the latter of which is roughly equivalent to MADRS ⩽10–11 (Zimmerman *et al.* 2004*c*).

Regarding the severity thresholds, the 32 studies that provided data are summarised in Tables 2–4.

### Severity threshold for the asymptomatic range

Studies focusing on the asymptomatic threshold could be roughly divided into three groups, reflecting differences in the used criteria for determining the ‘best’ threshold for the asymptomatic range.

The first group of studies selected the optimal threshold by maximising the correspondence to some gold standard (Hawley *et al.* 2002; Zimmerman *et al.* 2004*d*, 2005; Bandelow *et al.* 2006; Ballesteros *et al.* 2007; Riedel *et al.* 2010; Romera *et al.* 2011; Leucht *et al.* 2013; Sacchetti *et al.* 2015), most often the Clinical Global Impression-Severity scale (CGI-S) or some measure of functioning (see Table 2). The second group of studies based on the optimal asymptomatic threshold on the mean scores or statistical upper limits of the general population (Zimmerman *et al.* 2004*a*, *b*; see Table 3). These two groups mentioned a variety of optimal asymptomatic thresholds for the HAMD-17 ranging from ⩽2 (Zimmerman *et al.* 2005) to ⩽10 (Zimmerman *et al.* 2004*b*) and for the MADRS ⩽4 (Zimmerman *et al.* 2004*a*, 2004*d*) to ⩽11 (Bandelow *et al.* 2006).

The third and largest group of studies compared the prognosis of patients with different levels of depressive symptomatology, usually in terms of relapse/recurrence risk (see Table 4). Based on this information, a threshold can be chosen that best distinguishes those with a favourable from those with a bad prognosis, argued by Zimmerman *et al.* (2004*a*) to be the best method of validating a threshold. Most of these studies show that the presence of ‘subthreshold’ symptoms (often called residual symptoms if occurring after an MDD episode) was associated with an enhanced risk of a (recurrent) episode or relapse (Maier *et al.* 1997; Riso *et al.* 1997; Judd *et al.* 1998, 2000, 2016; Van Londen *et al.* 1998; Fava *et al.* 1999; Kanai *et al.* 2003; Taylor *et al.* 2004; Nierenberg *et al.* 2010; Dunlop *et al.* 2012; Kiosses & Alexopoulos, 2013; Peselow *et al.* 2015). One study (Romera *et al.* 2011) did not find this increased risk. Often authors implicitly argued for a lower threshold for remission that does not encompass this level of symptomatology. Some studies also showed that remission as defined by Frank *et al.* (1991), HAMD-17 ⩽7, is associated with a better prognosis than not achieving this level of remission (Paykel *et al.* 1995; Pintor *et al.* 2004).

Saliently, some other noteworthy studies showed a large discrepancy between Frank's definition of depression and patient's own judgement regarding their remission (Zimmerman *et al.* 2012*a*, *b*). Within the group of remitters as defined by Frank *et al.* (1991), a substantial heterogeneity was observed with respect to reported symptoms (Zimmerman *et al.* 2012*c*), psychosocial impairment (Zimmerman *et al.* 2004*e*, 2007) and a range of other relevant outcomes (Zimmerman *et al.* 2012*d*) (see Table 3).

### Severity threshold for the fully symptomatic range

Only one study focusing on the fully symptomatic threshold was obtained (see Table 2). By using the CGI-S of 2 or 3 as the gold standard, Leucht *et al.* (2013) advise a HAMD-17 threshold of ⩾7 or ⩾14, respectively.

### Duration threshold for episode

Frank *et al.* (1991) categorised the symptomatic period following any non-depressive state using a time boundary, separating the time period before the symptoms were recognised as part of a depressive episode from the time period afterwards. The underlying assumption was that developing transient depressive symptoms is not necessarily pathological, as long as they do not culminate in a long-lasting depressive episode. Regarding the validation of this duration criterion, Frank *et al.* (1991) state that an episode should be declared ‘*when it is unlikely that the patient will spontaneously recover in the next day or two*’. Although rather arbitrary, the concept is clear: for the validation of this duration criterion, data are necessary that shed light on the prognosis of those with recently started depressive symptomatology.

Such data was provided by four studies (see Table 5). The meta-analysis by Whiteford *et al.* (2013) covering the rate of spontaneous remission in untreated depression showed that this rate decreases continuously over time. However, the amount of data in the range of short duration of follow-up is rather scarce and the studied population (wait-list and primary care samples) is not representative of the general population with depressive symptoms.

One study in the general population showed that 25% of depressive episodes remitted after 4 weeks and 50% after 8–12 weeks, using a methodology in which onset and end of depressive episodes were retrospectively assessed by asking the respondents for their depressive symptomatology in the past (Eaton *et al.* 1997). The finding of a median duration of 12 weeks was replicated in the NEMESIS study using a similar methodology, which also shows that the rate of recovery quickly diminishes after these 12 weeks (Spijker *et al.* 2002).

Wakefield & Schmitz (2013) argued that ‘uncomplicated’ depressive episodes, defined as <2 months in duration combined with the absence of certain ‘heavy’ symptoms such as suicidal ideation and psychomotor retardation, should not be classified as MDD. They argued that the risk of developing new depressive episodes for those who had such an uncomplicated episode is not higher than for the general population. Thus, this subgroup of patients does not seem to suffer from an underlying disorder that increases their risk of developing subsequent depressive episodes. This suggests that, at least for this subgroup, the depressive symptomatology should be at least 2 months of duration before it should be considered as a depressive episode.

### Duration thresholds for remission and recovery

Frank *et al.* (1991) categorised the asymptomatic period following a fully symptomatic period with two time boundaries, yielding three distinct time periods: those (i) before the onset of full remission, (ii) following the onset of full remission but before declaration of recovery and (iii) after declaration of recovery. The underlying assumption is that these three successive periods are each associated with a certain ‘hazard’ for a return of symptoms, which diminishes significantly at each time boundary and becomes constant when recovery is declared.

In the available literature, the hazard for a return of symptoms for asymptomatic individuals is usually shown indirectly in the form of survival curves, showing the fraction of subjects without relapse/recurrence over time. An exponential survival curve is thus equivalent to a constant hazard, whereas a sudden decrease in a hazard (for example, when remission is achieved) should be visible as an upward discontinuity in the survival curve slope.

Survival curves (or equivalent) for asymptomatic individuals until relapse/recurrence or equivalent data were obtained from 31 studies (see Table 5). There is a substantial difference between studies in their studied populations (viz., general population, 1st, 2nd or 3rd line ambulant patients or inpatients), their operationalisations of remission, recovery, relapse and recurrence (because of different instruments or cut-offs on the same instruments) and in the involved treatments that are often uncontrolled.

Several studies show some indication of a sudden drop in relapse/recurrence rate a certain time after remission/recovery was obtained (Paykel *et al.* 1995; Riso *et al.* 1997; Judd *et al.* 1998; Van Londen *et al.* 1998; Heinze *et al.* 2002; Kanai *et al.* 2003; Kennedy *et al.* 2003; Naz *et al.* 2007; Holma *et al.* 2008; de Jonge *et al.* 2010; O'Leary *et al.* 2010; Kiosses & Alexopoulos, 2013). However, the exact amount of time necessary to achieve this drop (as counted from the start of the asymptomatic period) differs per study, ranging from about 2 months (O'Leary *et al.* 2010) to about 3 years (Judd *et al.* 1998). Other studies do not find such a sudden drop at all, instead suggesting that the diminishing hazard of return of symptoms is a gradual process rather than a discrete one (Maj *et al.* 1992; Shea *et al.* 1992; Flint & Rifat, 1997; Kessing *et al.* 1998; Van Weel-Baumgarten *et al.* 1998; Mueller *et al.* 1999; O'Leary *et al.* 2000; Solomon *et al.* 2000; Mattisson *et al.* 2007; Dunlop *et al.* 2012; Martínez-Amorós *et al.* 2012; Seemüller *et al.* 2014; Peselow *et al.* 2015; Judd *et al.* 2016). In particular, several studies of the long-term course of MDD show that recurrence rates stabilise only after many years, such as 2.5 years (Solomon *et al.* 2000), 10 years (Mattisson *et al.* 2007) or about 15 years (Kessing *et al.* 1998). A third group of studies shows atypical survival curves where the time-specific risk of return of symptoms even increases over time during certain time intervals (Eaton *et al.* 1997; Emslie *et al.* 1997; Birmaher *et al.* 2004; Pintor *et al.* 2004; Yiend *et al.* 2009).

## Discussion

### Severity thresholds

The obtained studies that aimed to identify the optimal thresholds for the asymptomatic and fully symptomatic depressive ranges differed widely in their methodologies (see Tables 2–4). Frank *et al.* (1991) postulated that these ranges should (i) correspond to what clinicians view as asymptomatic and fully symptomatic and (ii) that classification of patients within these ranges should be reasonably stable over time. Other theorists argued that the optimal thresholds should be selected based on their predictive value for the future course (Zimmerman *et al.* 2004*e*), which would be most consistent with methods used in other medical fields (Zimmerman *et al.* 2004*a*).

### Severity threshold for the asymptomatic range

Multiple studies showed that those who scored below a certain threshold on depressive symptom scales had a better prognosis than those who scored above it (Paykel *et al.* 1995; Maier *et al.* 1997; Riso *et al.* 1997; Judd *et al.* 1998; 2000, 2016; Van Londen *et al.* 1998; Fava *et al.* 1999; Kanai *et al.* 2003; Pintor *et al.* 2004; Taylor *et al.* 2004; Nierenberg *et al.* 2010; Dunlop *et al.* 2012; Kiosses & Alexopoulos, 2013; Peselow *et al.* 2015). Often this finding was presented as evidence for the perspective that the asymptomatic threshold is currently too high (Judd *et al.* 1998). However, even though none of these studies systematically studied and compared the predictive value of all possible thresholds, we hypothesise that this is a general finding that can be obtained irrespective of the chosen threshold, as a lower score on a depressive symptom scale increases the ‘symptomatic distance’ to the fully symptomatic threshold and therefore the average time required for reaching that state. Indeed, some studies show that the currently often-used threshold (HAMD-17 ⩽7; Frank *et al.* 1991) also differentiates in this regard (Paykel *et al.* 1995; Pintor *et al.* 2004). Studies using other methodologies for determining the best asymptomatic threshold – such as optimising correspondence to clinical impressions of clinicians (using the CGI-S as a gold standard), different functioning scales, or the general population – yield different optimal thresholds. The consensus among these authors seems to be that the currently often-used threshold of HAMD-17 ⩽7 is too high, as it leads to the inclusion of too many patients with poor functioning (Sacchetti *et al.* 2015), who are psychosocially impaired (Zimmerman *et al.* 2007) and who do not consider themselves as remitted (Zimmerman *et al.* 2012*a*).

Ultimately, the particular choice of asymptomatic threshold is rather arbitrary given the available evidence. Nonetheless, the currently often-used threshold seems to be too high. We, therefore, suggest lowering the asymptomatic threshold to ⩽4 on the HAMD-17; this is on the low side of the suggested values in the obtained studies – which we think is justified given the better functioning below this score (Sacchetti *et al.* 2015) – although still above the mean score in the general population (Zimmerman *et al.* 2004*b*). It has been shown that some patients who scored ⩽7 on the HAMD-17 still met diagnostic criteria for MDD (Zimmerman *et al.* 2004*e*), which is another argument for our suggestion to lower the asymptomatic threshold to ⩽4, as this largely prevents ‘remitted’ people from meeting the diagnostic criteria for MDD. This new HAMD-17 threshold is roughly equivalent to a threshold of ⩽5 on the MADRS (Mittmann *et al*. 1997), which is plausible given the reviewed evidence. Note that these thresholds are useful as endpoints in clinical studies, but do not necessarily mean that scoring below these thresholds should be the main treatment goal for clinicians, as treating individual patients by striving for the lowest score possible still improves prognosis (Taylor *et al.* 2004).

### Severity threshold for the fully symptomatic range

Only one study was obtained that provides some evidence for the fully symptomatic cut-off (Leucht *et al.* 2013). This relative lack of evidence is understandable, as this seems to be the definition that least ‘needs’ empirical validation; this can be understood as a rather subjective clinical decision regarding the minimal level of symptomatology that can be considered to be a disorder. Therefore, there is not enough evidence to make any recommendations regarding this threshold.

### Duration threshold for episode

Only a limited amount of studies showed data on the prognosis of those with ‘recent-onset’ depression (see Table 5). This can be explained by epidemiological investigations that typically include depressed populations, for which it is unclear how long the depressive symptoms have been present at the start of the studies. Although two studies show that half of the depressive episodes in the general population remit within 3 months after their onset (Eaton *et al.* 1997; Spijker *et al.* 2002), it seems likely that many short ‘episodes’ of only a few days are missed since these episodes are infrequently retrospectively indicated, and short episodes are more easily forgotten than long ones (Moffitt *et al.* 2010). Therefore, the rate of early remission is probably even higher than suggested by these studies.

In general, the reviewed data suggest that the rate of (spontaneous) remission of depressive symptoms is relatively high when the onset of these symptoms is recent, especially during the first 12 weeks, but diminishes quickly thereafter. This provides some justification for the suggestion by Frank *et al.* (1991) of requiring a certain amount of time at the fully symptomatic level before defining a depressive episode. However, the currently required ‘waiting time’ of only 2 weeks (see Table 1; DSM-5 criteria, APA, 2013; ICD-10 criteria, WHO, 1993) does not seem to be based on empirical evidence. The reviewed studies suggest that a longer time period might be advisable. Nonetheless, we refrain from a definitive conclusion, for which a prospective study in which the general population is screened with a high frequency (e.g. weekly) for depressive symptomatology is required but hitherto unavailable.

### Duration thresholds for remission and recovery

A substantial body of literature studying depressive relapse/recurrence risk over time has been obtained (see Table 5), but comparing the studies is not straightforward; the studies differed in their studied populations, their operationalisations of remission, recovery, relapse and recurrence, and in the involved treatments. Some studies were consistent with the idea of a ‘point of rarity’ (Frank *et al.* 1991) at which the relapse/recurrence risk suddenly drops or becomes stable. However, there is no consistency in the estimation of this time point. Combined with the fact that the majority of studies do not show such a point of rarity, the most likely conclusion is that prognosis gradually improves as remission/recovery duration is longer, rather than suddenly at a particular point in time.

The reviewed data do not suggest that any specific duration threshold to distinguish remission from recovery is warranted to add predictive value to the observation that prognosis improves over time as the duration of the asymptomatic period increases. Not only were the specific operationalisations of the duration criteria by Frank *et al.* (1991) and Rush *et al.* (2006) not empirically supported, it seems that the whole concept of these duration criteria must be rejected. The idea that a reoccurrence of depressive symptoms shortly after their initial remission constitutes a ‘relapse’ of the previous episode, whereas their later reoccurrence is the first sign of an entirely new episode, is a model that lacks empirical support. Additionally, it is of no additional value to the patient or clinician as the assumed origin of the reoccurring symptoms has no implications for treatment or prognosis.

Thus, based on these results, the duration criteria for declaring remission and recovery seem unnecessary. We suggest that depressive remission can simply be defined as the asymptomatic state after a depressive episode, without applying any duration criterion. Stability of remission is then relatively low on the first day but increases gradually with its duration. The term recovery can then be used as a concept that includes more than just absence of symptoms, such as social functioning or subjective well-being, possibly including the absence of significant treatment as this would better fit the concept of recovery from a patient's perspective.

### Limitations

Limitations of this review include the greatly varying study populations and treatments within the included studies (which is also a strength). Moreover, a substantial part of the data had to be extracted from survival curves that only rarely showed confidence intervals and often did not possess a clearly labelled time axis, making it difficult to assess exactly when the measurement began.

## Conclusions

More than a quarter-century after the landmark paper in which Frank *et al.* (1991) provided their consensus-based definitions for depressive states (episode, remission, recovery, relapse, recurrence), we reviewed the empirical evidence. The data suggest that remission can best be defined as a less symptomatic state than assumed earlier (HAMD-17 ⩽4 instead of ⩽7), without applying a duration criterion. Specific duration thresholds to separate remission from recovery are not meaningful. Evidence suggests that the minimal duration of depressive symptoms before a depressive episode can be defined should be longer than 2 weeks, although further studies are required to recommend an exact duration threshold.

## Supplementary material

For supplementary material accompanying this paper visit https://doi.org/10.1017/S2045796018000227. click here to view supplementary material

## Availability of data and materials

The data regarding the process of screening and selection of the articles included in this systematic review (after removal of identical articles) are available in an online supplement.
