# Supplementary material for: Empirical evidence for definitions of episode, remission, recovery, relapse and recurrence in depression: a systematic review
Source: Epidemiol Psychiatr Sci. 2018 May 17;28(5):544–62. doi: 10.1017/S2045796018000227 (PMC7032752; doi:10.1017/S2045796018000227)
Supplement: Supplementary file 1 [file S2045796018000227sup.zip › S2045796018000227sup001/Supplementary_table.docx]

Supplementary table. Conceptualizations of MDD episode, remission, recovery, relapse, recurrence by Frank et al. (1991)

| Episode | A period lasting ≥D days during which the patient is consistently within the fully symptomatic range. An episode ends only when the patients reaches recovery. |
| --- | --- |
| Remission (full) | A relatively brief period lasting >E days but <F days within or at the end of an episode during which the patient is consistently in the asymptomatic range. A remission can be followed by a relapse or a recovery. |
| Recovery | A period lasting ≥F days at the end of an episode during which the patient is consistently in the asymptomatic range. A recovery designates the end of an episode. |
| Relapse | A return of symptoms to the fully symptomatic range during the period of remission, but before recovery is attained. A relapse represents the return of symptoms of a still ongoing but symptomatically supressed episode. |
| Recurrence | A return of symptoms to the fully symptomatic range during the period of recovery. A recurrence represents an entirely new episode. |
