# Supplementary material for: Empirical evidence for definitions of episode, remission, recovery, relapse and recurrence in depression: a systematic review
Source: Epidemiol Psychiatr Sci. 2018 May 17;28(5):544–62. doi: 10.1017/S2045796018000227 (PMC7032752; doi:10.1017/S2045796018000227)
Supplement: Supplementary file 1 [file S2045796018000227sup.zip › S2045796018000227sup001/PRISMA_Flow-Diagram.doc]

**PRISMA 2009 Flow Diagram**

**Screening**

**Included**

**Eligibility**

**Identification**

Records identified through database searching
(n = 1570)

Records after duplicates and 26 non-obtainables removed
(n = 1330)

Records screened
(n = 1330)

Records excluded,

with reasons^1^
(n = 1213)

- No MDD (n=250)
- Not empirical (n=315)
- Study type not useful (n=411)
- Association or cause-effect study (n=237)

Full-text articles assessed for eligibility
(n = 166)

Additional records identified through other sources^2^
(n = 49)

Full-text articles excluded because of lack of relevant data^3^
(n = 110)

Studies included in qualitative synthesis
(n = 56)

^1^Records were excluded based on title and abstract if they *i)* did not focus on individuals with MDD, *ii)* were non-empirical, *iii)* were of study types not expected to yield relevant information for the purpose of this review (excluded are: treatment evaluations (including RCT’s), brain imaging/EEG studies, economic evaluations, psychometric evaluations not focusing on HAMD-17 or MADRS, sleep studies, studies involving genes, hormones or anything lab-related, case studies, survey/agreement studies, qualitative studies, methodological studies involving sample data, evaluations of prevention programmes and suicide studies), or *iv)* had the evaluation of some association or cause-effect relation between variables as the main goal (most studies excluded for this reason were examining risk factors for MDD or MDD as a risk factor for later functioning).

^2^All included articles were checked for other references that were considered likely to be relevant, published in 1991 or later and were not yet included.

^3^Full-text articles were more thoroughly assessed for presenting data that could be used for validation of at least one of Frank et al.’s (1991) definitions.
